# An Online Gamified Pain Management Program for Adults With Chronic Pain: Protocol for a Randomized Controlled Trial

**DOI:** 10.2196/80542

**Published:** 2026-01-13

**Authors:** Mun Yee Mimi Tse, Jiafan He, Tyrone Tai On Kwok, Sukki Ho

**Affiliations:** 1 School of Nursing and Health Sciences Hong Kong Metropolitan University Kowloon China (Hong Kong); 2 Hong Kong Polytechnic University Kowloon China (Hong Kong)

**Keywords:** pain, pain management, gamification, digital health, self-determination theory, randomized controlled trial

## Abstract

**Background:**

Although digital interventions have been widely used in chronic pain management, evidence on their effectiveness has demonstrated heterogeneity. They have low retention rates, which are exacerbated by a lack of feedback, social interaction, and engaging content, contributing to boredom and higher attrition. Gamification offers a possibility to overcome these barriers and enhance user motivation and engagement.

**Objective:**

This study aims to investigate the effects of an online gamified pain management program in a sample of adults with chronic noncancer pain.

**Methods:**

A 2-arm, single-blind randomized controlled trial will recruit 172 participants through Chinese hospitals and online platforms. Eligible participants will be randomized to 2 groups: a 10-week gamified pain management program for the experimental group and routine online pain management materials for the control group. Primary outcomes, including pain intensity and pain interference, and secondary outcomes, including fear avoidance, emotion, exercise adherence, quality of life, and user satisfaction, will be assessed at baseline, after the intervention (10 weeks), and at a 3-month follow-up. A mixed-design ANOVA and intention-to-treat analysis will be used to evaluate intervention effects.

**Results:**

The trial is registered, with recruitment ongoing from July 2025 to August 2026. A total of 172 participants (86 per group) will be recruited. As of November 17, 2025, we have recruited 19 participants. Results are expected to be published in March 2027

**Conclusions:**

The findings will address a critical gap by examining a novel gamified intervention within a theory-driven design to provide an engaging solution to improve self-management in people with chronic pain.

**Trial Registration:**

Chinese Clinical Trial Registry ChiCTR2400094247; https://www.chictr.org.cn/hvshowproject.html?id=266029&v=1.0

**International Registered Report Identifier (IRRID):**

DERR1-10.2196/80542

## Introduction

The Global Burden of Disease Study highlights the substantial prevalence and impact of pain globally [1]. Chronic pain, defined as pain persisting for more than 3 months, significantly affects various aspects of daily life, including work performance, physical activity, and social functioning. It is often associated with psychological disorders such as anxiety and depression, sleep disturbances, substance use, cognitive impairment, and reduced work productivity [2].

Existing guidelines advocate for biopsychosocial treatments that incorporate education, exercise, and psychological interventions to reduce pain in both the short and long term. These approaches emphasize the importance of physical activity and engagement in meaningful daily activities [3,4]. However, the International Association for the Study of Pain has concluded that the training provided to health professionals in pain management is insufficient to achieve effective outcomes [5]. Additionally, patients with chronic pain often face substantial challenges in maintaining physical activity, as they may feel both physically inactive and emotionally hesitant due to the fear of exacerbating pain [6,7].

Online platforms have revolutionized the management of chronic pain by providing accessible alternatives to conventional medical treatments [8]. These platforms allow patients to engage with pain management strategies at their own pace, overcoming barriers such as geographic isolation and socioeconomic disadvantage [9]. Studies suggest that telerehabilitation saves costs and that web-based exercise interventions improve home exercise adherence and confidence [10,11]. Despite their potential, existing online pain management programs often suffer from low retention rates, a problem that is exacerbated by a lack of feedback, social interaction, and engaging content [12]. Gamification has emerged as a promising solution to these challenges, offering an approach that can enhance user engagement by incorporating game elements that motivate and sustain participation [13].

Gamification leverages game mechanics to influence user behavior, improving engagement, motivation, and overall health outcomes [14]. In health care, gamification has proven effective in addressing various health issues and promoting physical activity by making exercise and learning both enjoyable and motivating [15,16]. The use of motivational affordances within gamification has been shown to positively influence psychological factors, such as motivation, attitude, and enjoyment [17], making it a potentially powerful tool for behavior change. Regarding chronic pain, a systematic review demonstrated that gamified exercise interventions, including those using platforms such as Kinect Sport, Nintendo Wii, and virtual reality, can effectively reduce pain by providing interactive scenarios and rewards [18].

Despite these benefits, existing research has not fully explored gamification within the biopsychosocial framework for chronic pain management. Most studies have focused primarily on increasing physical activity, while neglecting the critical educational and psychological components that are essential for comprehensive pain management [18]. As fear of pain is associated with avoidance behaviors, educational interventions, such as those based on pain neurophysiology, can help patients better understand pain and modify their attitudes toward it, potentially leading to improved outcomes [19]. The ability of patients to accept pain sensations, rather than attempting to fight them, has been associated with greater reductions in pain interference [20]. Recent studies have also highlighted the effectiveness of biopsychosocial interventions that combine physical exercise and psychological support to reinforce positive behaviors and challenge negative pain-related perceptions [21,22].

To address this gap in the literature, an online gamified pain management program, based on an escape room task, will be developed and augmented with game elements. The aim of this study is to investigate the effects of online gamified pain management in a sample of adults with chronic noncancer pain via a randomized controlled trial. To this end, the objectives will be to (1) examine the effects of the gamified pain management intervention on the levels of pain intensity, pain interference, psychological outcomes, and quality of life among adults; (2) assess exercise adherence and game experience in the gamified home-based management program; and (3) identify the relationships between the effects of the intervention and sociodemographic factors.

## Methods

### Study Design and Setting

This study is a 2-arm, single-blind (analysts are blinded), randomized controlled trial. A total of 172 individuals with chronic pain will be recruited and randomly assigned to either gamified pain management program (intervention group) or a digital resource package (control group). The interventions for both groups will be delivered online. Participants will complete outcome assessments at 3 time points: baseline (T0), immediately after the intervention (T1), and at a 3-month follow-up (T2). This study protocol follows the SPIRIT (Standard Protocol Items: Recommendations for Interventional Trials) guidelines [23].

### Sample Size

A previous power analysis was used for sample size calculations using G*Power software (version 3.1; Heinrich-Heine-Universität Düsseldorf) [24]. To establish the effect size for the sample size calculation, we synthesized evidence from multiple sources. A meta-analysis analyzing the effects of serious games on pain intensity in patients with chronic pain reported a mean difference of −0.62 (95% CI −1.15 to −0.10) for pain reduction [18]. To supplement this, we conducted another meta-analysis of 12 relevant studies examining the effects of gamification on pain intensity in adults. The results yielded a mean difference of −0.86 (95% CI −1.61 to −0.11) [25-36]. However, given that our digital intervention comprises mixed physical, psychological, and educational components, we also incorporated evidence from 2 other meta-analyses including online pain management and multidisciplinary programs, with effect size of −0.30 (95% CI −0.50 to −0.10) [12] and −0.57 (95% CI −0.63 to −0.50) [37].

Therefore, to ensure robust and conservative statistical power, we selected a medium effect size of 0.50 to reduce potential bias or heterogeneity [38]. The following parameters were used: power (1−β)=0.90, effect size=0.50, and α=.05 (2 tailed). Taking a 20% margin for dropout and missing data, a total of 172 participants will be recruited, with 86 allocated to the experimental group and 86 to the control group.

### Participants and Recruitment

Participants recruitment will be conducted through both on-site and online approaches. A series of offline educational lectures accompanied by recruitment flyers or posters will be held at outpatient pain clinics at Tianhe District People’s Hospital and community service centers in Guangdong province to attract potential participants. Online recruitment will be conducted via social media platforms, including WeChat (version 8.0.66; Tencent Holdings Limited), Xiaohongshu (version 9.14; Xingyin Information Technology Limited), and TikTok (version 6.9.0; ByteDance), by posting advertisements. All recruitment materials will include a brief introduction to the study aim, implementation process, and a survey hyperlink on the Wenjuanxing platform [39] (Ranxing Information Technology Co, Ltd), which will direct participants to the consent form and eligibility screening.

To be eligible to participate in the study, the participants must meet all the following inclusion criteria: (1) be aged 18 years or older, (2) be able to read and understand Mandarin Chinese, (3) own a smartphone with internet access, (4) have a previously diagnosed chronic noncancer pain condition lasting more than 3 months [40], and (5) report a score of ≥2 on the visual analogue scale [41].

Participants will be excluded if they meet one or more of the following exclusion criteria: (1) associated pathologies that make it impossible to perform physical exercise (eg, myopathies, neurological diseases, cardiac diseases, pregnancy, pulmonary diseases, infection, or fracture) [42], (2) undergoing surgery or invasive treatments within the past 3 months [43], or (3) concurrent participation (or participation within the preceding 3 months) in a supervised exercise program or multidisciplinary treatment.

### Procedure and Randomization

The eligibility screening survey will automatically classify individuals after clicking the Agree button as they agree to provide consent. Screening questions will consist of pain intensity, pain duration, health history, recent treatment enrollment, and demographic data. The survey will be designed with conditional logic such that individuals who do not meet the eligibility criteria will be automatically redirected to the end of the survey and prompted to submit their responses. This approach means individuals who are not eligible will neither complete the full survey nor engage in the intervention. Conversely, eligible individuals will proceed to complete the remaining sections, which constitute the full baseline assessment. At the end of the questionnaire, interested participants may provide their WeChat account to receive group allocation and intervention information.

Following baseline assessment, enrolled participants will be randomly assigned in a 1:1 ratio to either the intervention or the control group. A computer-generated randomization sequence using blocks of sizes 2 and 4, stratified by age and sex, will be used. The randomization process will be managed by an independent researcher (JH), and allocation numbers will be stored securely in a password-protected database. Allocation will be revealed to the intervention deliverer (Chen Z, a PhD student) only after a participant is enrolled. The intervention deliverer will be blinded to data assessment and data analysis.

After randomization, participants will be notified of their group allocation and provided with preassigned WeChat log-in credentials. Those in the intervention group will receive a direct message containing a hyperlink to access the gamified pain management platform, while those in the control group will receive a digital resource package. After the intervention, T1 (10 weeks immediately after intervention) and T2 (3-month follow-up) assessments will be collected via the Wenjuanxing platform [39], and semistructured interviews will be conducted to collect individual’s perceptions and experiences of the intervention. Participants who report the most, moderate, or least change in pain intensity will be invited to participate in the interviews.

### Study Intervention

The design of the gamified pain management is based on established gamification principles and frameworks [44,45] and is theoretically grounded in self-determination theory (SDT), which supports individual autonomy, competence, and relatedness to improve intrinsic motivation and sustain engagement [46]. The intervention was co-designed by an expert in chronic pain (MYMT) and an expert in digital health (TTOK), with contributions from the lead researcher (JH), a pain clinic clinician, a physiotherapist with more than 5 years of experience, and 3 internet technology specialists. The intervention is delivered via the web-based platform VxEditor [47], which is accessible on both smartphones and computers, over a maximum of 10-week period, allowing participants to complete the program at their own pace. In addition, participants can choose their specific pain site via the platform, which tailors exercises accordingly and thus improve autonomy.

The gamified pain management is designed as an “escape room” game. Participants assume the role of a hero (avatar) guiding others to escape from a metaphorical “pain room,” which fosters a sense of relatedness. The intervention consists of 8 core “rooms” (sessions). Participants must complete various challenges, including unlocking 10 clues and accumulating at least 60 points to progress from one session to the next, which is designed to foster a sense of competence. Participants are awarded 5 points for completing each exercise task and 2 points for correct quiz answers, while skipping an exercise earns no points. To further support competence and reinforce the knowledge of pain, immediate and automatic feedback is provided, offering affirmation and encouragement for correct quiz answers and explanatory feedback for incorrect answers. Four optional hidden sessions are also available, offering additional challenges for highly engaged users. A detailed overview of the gamification design is provided in Table 1.

**Table 1:** Gamification design of the interventions.

Game elements	Applicable gamified interventions
Anonymity	Users are anonymized and represented by a unique code.
Avatar	Users assume the role of a hero (avatar) in the game.
Narrative	A background story (escape from the pain room) is presented to engage users.
Tutorials	Introductory tutorials establish an emotional connection with users and provide step-by-step instructions.
Choices	Users can select different games based on their specific pain site.
Feedback	Users receive notifications upon completion of exercises and correct quiz answers.
Points	Users earn points for completing exercises and quizzes and for progressing to the next sessions.
Progression and leaderboard	A progress number indicates the level of effort achieved.
Challenges and rewards	Completing challenges unlocks hidden games with additional exercise opportunities.

The contents of the intervention consist of exercises complemented with education and relaxation practices, which are identified as “clues” within each session. Education topics are presented through animated videos and written notes, focusing on pain mechanisms, neurobiology and neurophysiology, the biopsychosocial model of pain, and management strategies [48]. Quizzes are used to assess and reinforce knowledge from the educational content. A total of 28 prerecorded exercise videos demonstrate stretching, strengthening, balance, and resistance exercises [49], tailored to specific pain areas (eg, neck, shoulder, back, knee, or head). The program recommends at least 60 minutes of exercise per week, and the completion of each exercise is verified via an in-video dialogue prompt that appears midway through the video, which participants must click to earn points. The relaxation practices are presented through recorded audios that guide participants in breathing exercises and muscle relaxation [50]. In addition, participants are required to solve real-world case puzzles by applying their knowledge to determine the mechanisms of chronic pain, which is a key task for session completion. Details of these components are shown in Table 2.

**Table 2:** The gamified pain management intervention components.

Session	Clue components
1	Exercise: three 6-minute balancing exercises Educational information: pain mechanisms Relaxation audio: breathing exercises Quiz and puzzle
2	Exercise: three 6-minute stretching exercises for different pain sites Educational information: etiology of pain Relaxation audio: breathing exercises Quiz and puzzle
3	Exercise: three 6-minute core strengthening exercises Educational information: neurobiology and neurophysiology of pain Relaxation audio: muscle relaxation Quiz and puzzle
4	Exercise: three 6-minute stretching exercises for different pain sites Educational information: biopsychosocial model of chronic pain Relaxation audio: muscle relaxation Quiz and puzzle
5	Exercise: three 6-minute core strengthening exercises Educational information: attention system and pain and distraction from pain Relaxation audio: body scan Quiz and puzzle
6	Exercise: three 6-minute balancing exercises for different pain sites Educational information: medication use and drug abuse Relaxation audio: body scan Quiz and puzzle
7	Exercise: three 6-minute core strengthening exercises Educational information: physical activity for chronic pain Relaxation audio: relaxation music Quiz and puzzle
8	Exercise: three 6-minute resistance exercises Educational information: nonpharmacological strategies Relaxation audio: outdoor relaxation Quiz
Four hidden sessions	Exercise: three 20-minute stretching and resistance exercises

**Figure 1:**
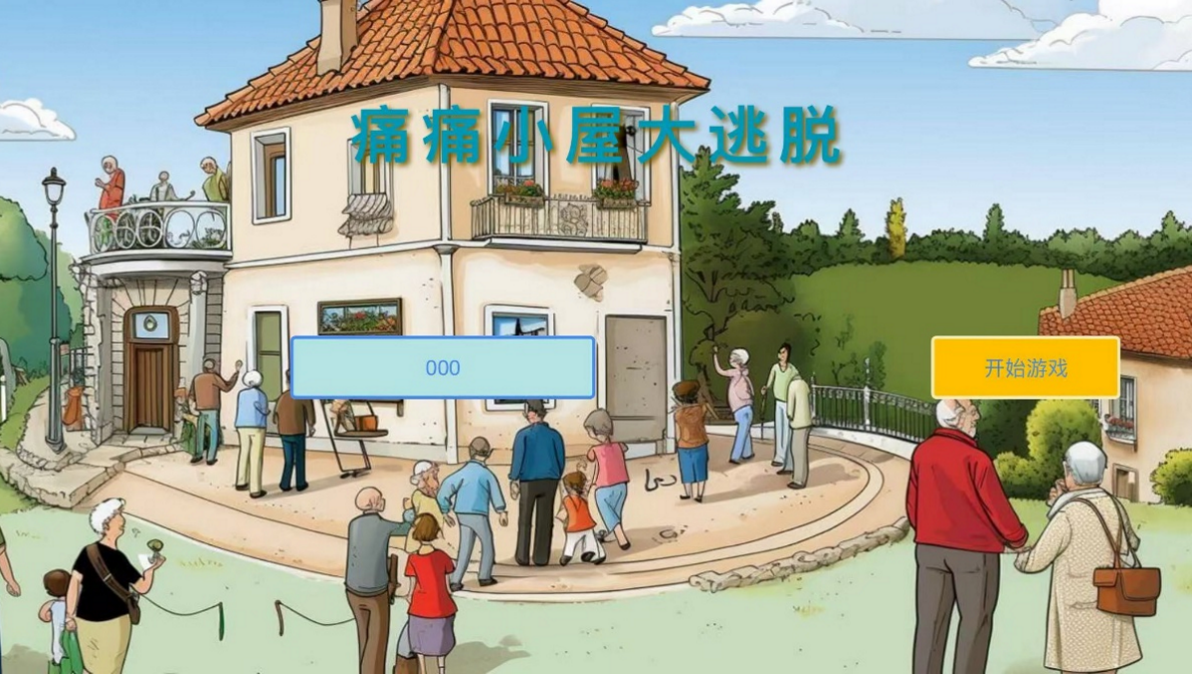
Home page interface of the gamified pain management intervention.

**Figure 2:**
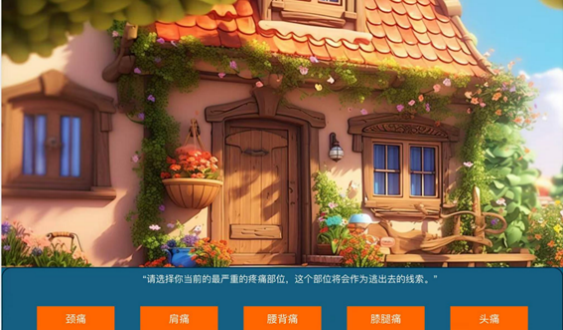
Choice feature interface.

**Figure 3:**
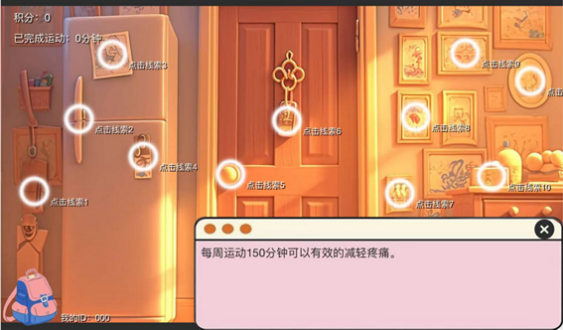
Room background and points feature interface.

**Figure 4:**
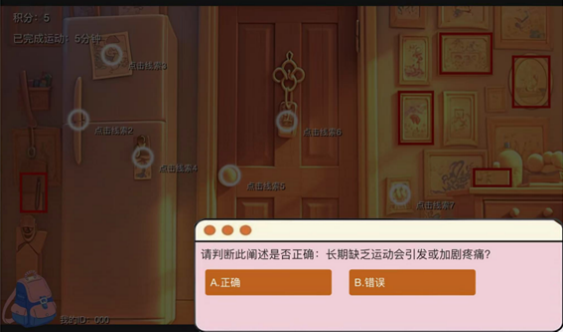
Quiz function interface.

The gamified pain management platform automatically captures and stores detailed engagement data on a secure, cloud-based back end. The latest room successfully completed is recorded, allowing research staff to track progress. Time stamps for the first log-in and last interaction with the platform are also generated to calculate the total time spent. Quiz accuracy rates and total points accumulated are stored on the backend, enabling monitoring by the research team. To promote adherence, interim reminders will be sent via WeChat biweekly to participants who have not progressed at the expected pace. After completing the program, participants are encouraged to continue using the platform for practice. Screenshots of the gamified pain management are provided in Figures 1-4.

### Control Group

In the control group, participants will receive a digital pain management resource package for a 10-week period, containing the same components as the gamified pain management group but delivered in a nongamified, standard digital format. The package contains educational content, exercise videos, educational videos, and relaxation audios that are identical in content and volume to those used in the gamified pain management group, delivered via an online PDF handbook with hyperlinks through WeChat. These multimedia resources are hosted on WeChat and the Xiaohongshu official account to ensure accessibility. In addition, participants will receive interim reminders biweekly via WeChat, encouraging them to read the handbook and engage in exercises, relaxation, and educational activities to promote adherence throughout the intervention period.

### Outcome Measures

The T0 (baseline before intervention), T1 (immediately after intervention), and T2 (the 3-month follow-up) assessments will be collected via the Wenjuanxing platform [39].

#### Primary Outcome

Pain intensity and pain interference are assessed using the Brief Pain Inventory between baseline, T1, and T2, rated from 0 (no pain) to 10 (worst pain). Pain severity is calculated based on pain experienced in the past 24 hours and “current pain.” Pain interference includes the degree to which pain interferes with general activity, mood, walking, work, social relationships, sleep, and enjoyment of life. The Chinese version has good reliability, with Cronbach α=0.89 [51].

#### Secondary Outcomes

##### Study Instruments

The Fear Avoidance Beliefs Questionnaire is used to measure fear of pain and consequent avoidant behaviors related to physical activity. The questionnaire consists of 16 items; each rated on a 7-point Likert scale ranging from 0 (completely disagree) to 6 (completely agree). The Cronbach α of the Chinese version ranges from 0.75 to 0.85 [52].

The Pain Catastrophizing Scale is used to measure pain catastrophizing beliefs, including 3 subscales, rumination, magnification, and helplessness, rated from 0 to 4. Higher scores indicate stronger pain catastrophizing beliefs. The Cronbach α of the Chinese version is 0.91 [53].

The Godin-Shephard leisure-time physical activity questionnaire is used to measure exercise adherence, including frequency of strenuous, moderate, and mild exercise per week. The percentage of exercises completed out of the total number of prescribed exercises is used to measure total exercise adherence [54].

The Patient Health Questionnaire-9 is used to assess the symptoms of depression, scored on a 4-point Likert scale from 0 (not at all) to 3 (nearly every day). Higher scores indicate greater severity of depressive symptoms. The Cronbach α of the Chinese version of this questionnaire is 0.86 [55].

The Generalized Anxiety Disorder-7 is used to measure anxiety, including 7 items rated from 0 (not at all) to 3 (nearly every day). The Chinese version has excellent internal reliability, with a Cronbach α of 0.91 [56].

The 3-level version of the EuroQol-5D is used to assess an individual’s perceptions of quality of life, including mobility, self-care, usual activities, pain and discomfort, and anxiety and depression. Each dimension is evaluated with 3 levels of severity. Higher scores indicate a lower level of quality of life. The Chinese version has been treated as the standard in China [57].

The Game User Experience Satisfaction Scale-18, an 18-item scale is used to evaluate overall user satisfaction with the game, rated from 0 (strongly disagree) to 7 (strongly agree), with good internal reliability (Cronbach α=0.93) [58]. Higher scores indicate a higher level of satisfaction.

##### Intraintervention Variables (Collected During or Immediately After the Intervention)

Engagement metrics are assessed through session-specific satisfaction scores (Satisfaction scores for sessions 1 to 8) and quiz accuracy, recorded via the gamified pain management platform. Satisfaction scores are derived from the sum of 3 items rated on a 5-point Likert scale following the completion of each session. These items evaluate the following: (1) understandability (“Are the materials in this session easy for you to understand?”), (2) comprehensibility (“How much do you understand the materials in this session?”), and (3) applicability (“To what extent do you think you would apply this session to future self-management?”). Quiz accuracy is calculated as the proportion of correctly answered questions in each session’s embedded quiz.

Treatment duration is automatically calculated as the total number of weeks between a participant’s first and last log-in on the gamified pain management platform.

##### Semistructured Interviews

Semistructured interviews are conducted by JH after completion of the intervention to measure participants’ perceptions, attitudes, and experiences after participating in the intervention. Interviews will be conducted via a private online Tencent Meeting room (version 3.37.10.401; Tencent Meeting) [59], ensuring that participants’ confidentiality and privacy are protected. In addition, perceived motivators and barriers related to gamification elements will be explored. Each interview will last 40 to 60 minutes. Examples of semistructured interview questions are provided in Textbox 1.

**Examples of interview questions.**

1. How has your pain intensity changed since the start of the gamified pain management intervention?
2. What changes have occurred in your perception of pain since you started receiving the gamified pain management intervention?
3. What aspects of gamified pain management do you think are most beneficial? How about the game elements?
4. What challenges or difficulties have you encountered when using the gamified pain management intervention?
5. Which game element do you find most useful, and why?
6. Do you plan to continue using the GPM intervention after this study? If not, what barriers are preventing you from continuing?
7. What suggestions do you have for improving the gamified pain management intervention?

### Statistical Analysis

SPSS (version 25.0; IBM Inc) will be used for all statistical analyses. An intention-to-treat analysis will be used, whereby all participants will be analyzed in the context of the groups to which they had been originally assigned. Descriptive statistics for feasibility will include the following: (1) the percentage of participants recruited from the total number approached; (2) the percentage of participants (among those recruited) who are retained in the study at the end of 3 months in each arm and overall; and (3) engagement metrics, including completion status, duration of the intervention, and rate of accuracy on quizzes.

To detect potential differences in sociodemographic and baseline clinical characteristics, the *χ*^2^ test with continuity correction (or the 2-sided Fisher exact test when appropriate) will be used for categorical variables, and the *t* test will be used for continuous variables. The normality of distributions will be confirmed using the Shapiro-Wilk test before statistical analysis. Repeated measures ANOVA (mixed design: within and between groups) will be used to determine the overall effectiveness of the intervention. Missing values will be addressed using intention-to-treat principles with maximum likelihood estimation, assuming data are missing at random. Statistical significance will be set at *P*<.05.

The interviews will be transcribed verbatim and reviewed. Open and axial coding will be used for data identification and integration, resulting in different meaning categories derived using grounded theory methods. NVivo (QSR International Inc) will be used for the coding process and information handling.

### Ethical Considerations

This study has been carried out in accordance with the guidelines of the Hong Kong Metropolitan University Research Ethics Committee (HE-SF2024/34) and was registered in the Chinese Clinical Trial Registry (ChiCTR2400094247).

All potential participants will be provided with a detailed digital informed consent form. This form outlines the purpose of the study, procedures, potential risks and benefits, confidentiality measures, voluntary participation, the right to withdraw at any time without penalty, and contact information for the principal investigator. Participants will be required to click the Yes button below the statement “I have read and understood the above information, and I voluntarily agree to participate in this study” to indicate their consent to participate.

To ensure privacy and confidentiality, all data will be anonymized by assigning a unique reference number, which will be used to link all data collected from the game and surveys, rather than using participants’ names. All data will be securely stored in an encrypted folder, with backups stored on a separate encrypted external hard drive to further protect participant confidentiality. Only the research team will have access to the information.

During the design of this study, care was taken to ensure that participants would not be exposed to any physical harm or discomfort. To prevent potential harm, all participants will be required to undergo prescreening to assess any existing health conditions that might contraindicate physical activity, as advised by their health care providers. Participants with such conditions will be deemed ineligible to participate in the study.

## Results

### Study Timeline

**Figure 5:**
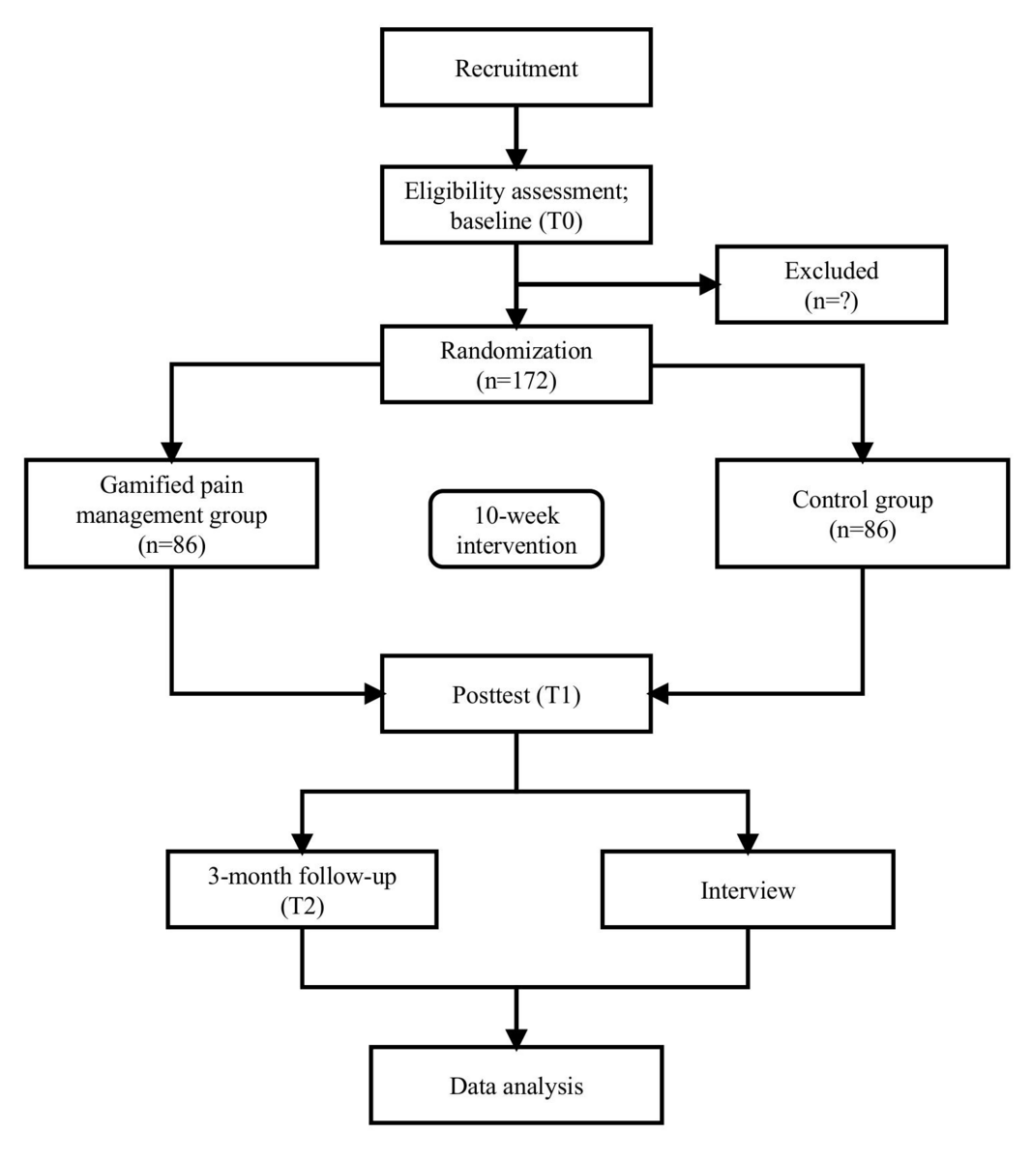
Trial flow diagram.

Recruitment was initiated in July 2025, and enrollment is ongoing. As of November 17, 2025, we have recruited 19 participants. Data analysis has not yet started. Final data collection is expected to be completed by August 2026, and the results are expected to be published in March 2027. Figure 5 shows the trial flow diagram.

### Anticipated Findings

We hypothesize that participants in the gamified pain management intervention will report a substantial reduction in pain intensity and pain interference compared with the control group at both postintervention and 3-month follow-up. Furthermore, the gamified pain management group is expected to exhibit greater improvements in secondary outcomes, including emotional distress, fear-avoidance beliefs, and pain catastrophizing, as well as higher engagement and exercise adherence. In addition, recovery is expected to be associated with sex, age, and socioeconomic status.

## Discussion

### Principal Findings

This study has demonstrated how gamification can be applied in health care. Gamification has been explored in various studies targeting different populations with diverse health goals. For example, a study conducted in 2024 on the use of virtual reality exergames in pain management showed positive effects for individuals with chronic lower back pain [60]. Given the insufficient adherence and motivation in chronic pain management, this study goes beyond the existing state of the art by developing a gamified pain management intervention, which reinforces patients’ motivation for self-management through an engaging approach combined with real-time feedback on achievements.

In the gamified pain management intervention, we will collect data through log-in time points, quiz responses, points accumulation, and weekly question-and-answer sessions on the gamified pain management platform. The information obtained is used to personalize physical exercises according to participants’ pain locations, thereby enhancing engagement. In addition, the intervention enhances user engagement and motivation by leveraging key principles of SDT. The escape room narrative and avatar role fulfill the need for relatedness by creating an immersive, purpose-driven experience that connects users to a larger mission. The points system, progress tracking, and tiered challenges address competence by providing clear goals, immediate feedback, and achievable milestones that validate skill development. Flexible pacing, pain-specific exercise choices, and optional hidden rooms support autonomy, allowing users to tailor the intervention to their preferences and capabilities. Together, these game mechanics, reinforced by educational content and physical practices, transform pain management into an intrinsically motivating process, consistent with studies demonstrating a moderate fit between SDT and the promotion of exercise and peer support for patients with chronic pain [61]. Furthermore, in the context of gamification, SDT provides a valuable lens for understanding how gamification elements influence motivation and sustain behavior change [62].

This randomized controlled trial was specifically designed to address key questions in the field of gamification research, particularly regarding the efficacy of gamification in pain management and the optimal design of such interventions. To our knowledge, this is the first randomized controlled trial combining gamified designs with education, exercise, and relaxation techniques aimed at chronic pain reduction. Randomization was implemented to prevent selection bias and control for confounding variables. Furthermore, while most existing studies focus primarily on pain intensity outcomes, this study incorporated psychological, perceptual, and satisfaction-related outcome measures to provide a more comprehensive evaluation of the intervention’s effectiveness.

### Limitations

Several limitations of this study warrant discussion. First, the research was conducted exclusively among adults, which may limit the generalizability of the findings. Future studies should aim to expand the applicability of the results by examining the impact of gamification on other populations, such as children, and across various settings. Second, at the time of designing this study, social interaction features were not incorporated into the gamified intervention. Given that loneliness is a common issue among individuals with chronic pain [63], the absence of a social context may have hindered the potential development of social and emotional skills and reduced opportunities to mitigate social isolation. Future research should explore the role of social interaction within gamification to determine whether it enhances engagement and outcomes.

In addition, although a click function was integrated into the exercise videos to monitor participant adherence, this method has limitations. Peer collaboration could potentially offer more benefits in terms of increasing participant engagement and adherence to the intervention. Future studies might consider incorporating more interactive elements, such as peer interactions, to improve the overall effectiveness of the gamified approach. All outcomes rely on self-reported measures or platform-collected data. As a pragmatic trial, medical diagnoses and pain medication details were not clinically verified, which may introduce reporting bias. Participants were recruited online, potentially favoring technologically adept individuals.

Finally, the distribution inequities of digital health care between rural and urban areas [64] and between younger and older adults in China limit the broader feasibility of this study, especially regarding the acceptability of digital games among older adults. Future studies should consider supporting initiative and confidence while preserving intrinsic capacity among older adults and individuals with digital illiteracy [65,66] by providing on-site training and multiple resources, including video-, paper-, or audio-based materials on technology use. These factors may limit generalizability to populations with lower digital literacy or limited internet access. In addition, due to the inherently visible nature of gamified interventions, participants in the intervention group would inevitably recognize their exposure to the gamified program, which may introduce expectation bias or placebo effects.

### Conclusions

This study aims to determine the impacts of online GPM on adults’ pain functioning and well-being. By leveraging a rigorous single-blind, randomized controlled trial design with standardized digital delivery, we provide a framework for assessing the efficacy of gamification in chronic pain management. Our findings may offer critical evidence on how gamified interventions can enhance engagement, adherence, and clinical outcomes, potentially informing future digital health strategies for chronic pain. Furthermore, the implementation of a fully remote trial demonstrates the feasibility and scalability of virtual interventions while ensuring accessibility for diverse populations. Ultimately, this research may help establish gamification as an effective tool for improving self-management in chronic pain, with implications for both clinical practice and digital therapeutic development.

## Abbreviations

SDT: self-determination theory
SPIRIT: Standard Protocol Items: Recommendations for Interventional Trials

## Data Availability

Data sharing is not applicable to this article as no datasets were generated or analyzed during this study.
